# Benefits of *Bifidobacterium breve* M-16V Supplementation in Preterm Neonates - A Retrospective Cohort Study

**DOI:** 10.1371/journal.pone.0150775

**Published:** 2016-03-08

**Authors:** Sanjay K. Patole, Shripada C. Rao, Anthony D. Keil, Elizabeth A. Nathan, Dorota A. Doherty, Karen N. Simmer

**Affiliations:** 1 Department of Neonatal Paediatrics, King Edward Memorial Hospital for Women, Perth, Australia; 2 Department of Neonatal Paediatrics, Princess Margaret Hospital for Children, Perth, Australia; 3 PathWest Laboratory Medicine Western Australia, Perth, Australia; 4 Women and Infants Research Foundation, King Edward Memorial Hospital for Women, Perth, Australia; 5 School of Women's and Infants' Health, University of Western Australia, Perth, Australia; 6 Centre for Neonatal Research and Education, University of Western Australia, Perth, Australia; Hôpital Robert Debré, FRANCE

## Abstract

**Background:**

Systematic reviews of randomised controlled trials report that probiotics reduce the risk of necrotising enterocolitis (NEC) in preterm neonates.

**Aim:**

To determine whether routine probiotic supplementation (RPS) to preterm neonates would reduce the incidence of NEC.

**Methods:**

The incidence of NEC ≥ Stage II and all-cause mortality was compared for an equal period of 24 months ‘before’ (Epoch 1) and ‘after’ (Epoch 2) RPS with *Bifidobacterium breve* M-16V in neonates <34 weeks. Multivariate logistic regression analysis was conducted to adjust for relevant confounders.

**Results:**

A total of 1755 neonates (Epoch I vs. II: 835 vs. 920) with comparable gestation and birth weights were admitted. There was a significant reduction in NEC ≥ Stage II: 3% vs. 1%, adjusted odds ratio (aOR) = 0.43 (95%CI: 0.21–0.87); ‘NEC ≥ Stage II or all-cause mortality’: 9% vs. 5%, aOR = 0.53 (95%CI: 0.32–0.88); but not all-cause mortality alone: 7% vs. 4%, aOR = 0.58 (95% CI: 0.31–1.06) in Epoch II. The benefits in neonates <28 weeks did not reach statistical significance: NEC ≥ Stage II: 6% vs. 3%, aOR 0.51 (95%CI: 0.20–1.27), ‘NEC ≥ Stage II or all-cause mortality’, 21% vs. 14%, aOR = 0.59 (95%CI: 0.29–1.18); all-cause mortality: 17% vs. 11%, aOR = 0.63 (95%CI: 0.28–1.41). There was no probiotic sepsis.

**Conclusion:**

RPS with *Bifidobacterium breve* M-16V was associated with decreased NEC≥ Stage II and ‘NEC≥ Stage II or all-cause mortality’ in neonates <34 weeks. Large sample size is required to assess the potential benefits of RPS in neonates <28 weeks.

## Introduction

Necrotising enterocolitis (NEC) continues to have significant mortality and morbidity including long-term neurodevelopmental impairment in very preterm neonates with gestation <32 weeks [1,2]. The outcomes are worse if surgical intervention is required, especially in extremely preterm neonates with gestation <28 weeks [3]. Despite decades of research, the pathogenesis of NEC is still not clear [4–6]. Excessive intestinal inflammatory response from an immature innate immune system and toll like receptors (TLR4) are currently considered to play an important role in its pathogenesis [7–10]. Having had no success in developing effective strategies for prevention of preterm birth, there have been limited options to reduce the risk of NEC. These included antenatal glucocorticoids, postnatal early and preferential breastmilk feeding, and standardised feeding protocols to minimise variations in feeding practice that have been epidemiologically linked to NEC [11–14].

Probiotics are live microorganisms that when administered in adequate amounts, confer benefits to the host [15]. Systematic reviews of randomised controlled trials (RCT) have shown that probiotics reduce the risk of NEC (≥ Stage II) and all-cause mortality significantly and facilitate enteral feeding in preterm very low birth weight (VLBW) neonates [16–21]. None of the trials reported adverse effects such as probiotic sepsis. There is broad consensus that probiotic effects are strain-specific [22–24]. Therefore despite the results from various meta analyses there has been a reluctance to adopt this intervention considering the heterogeneity of probiotic strains and protocols, population characteristics, type of feeds (milk/formula) and the trial settings [25–30]. However experts point out that clinical data to support strain-specific effects of probiotics are limited and the consistently decreased risk of NEC in RCTs using variable probiotic regimens suggests protection by different strains by shared beneficial pathways [31–33]. The number of reports on routine probiotic supplementation (RPS) indicates that clinical practice is changing in favour of probiotics in preterm neonates [34].

Ours is one of the largest neonatal intensive care units in the southern hemisphere (30 level III and 70 level II beds) that annually admits ~500 neonates with gestation <34 weeks including 100 to 120 with gestation <28 weeks. Considering the evidence in totality we decided to introduce RPS with *Bifidobacterium breve* M-16V (*B*. *breve* M-16V) for preterm neonates <34 weeks’ gestation in our unit. Both, the evidence supporting the use of this product in preterm neonates, and the results of our independent assessment of its quality including effect on fecal bifidobacteria, have been reported earlier [35].

### Aim

We aimed to assess if RPS with *B*. *breve* M-16V was associated with reduced incidence of NEC ≥ Stage II in preterm neonates born <34 weeks’ gestation [36].

### Hypothesis

We hypothesised that introduction of RPS would significantly reduce NEC ≥ Stage II [36].

## Materials and Methods

This was a retrospective cohort study comparing data from before (Epoch I: December 2008 to November 2010, n = 835) versus after (Epoch 2: June 2012 to May 2014, n = 920) introducing RPS with *B*. *breve* M-16V (Morinaga Milk Industry Co., Ltd, Japan). The data from the *B*. *breve* M-16V trial period between the two epochs was excluded [35].

### Ethics considerations

The study was approved by the Research Governance Committee, Women and Newborn Health Service (WNHS), Western Australia, based at King Edward Memorial Hospital for Women. Approval was also obtained from the Therapeutic Goods Administration (TGA, Canberra), under the Authorised Prescriber Pathway [37]. Written informed parental consent was obtained in the format approved by the WNHS Research Governance Committee and TGA, Canberra, Australia.

### Eligibility criteria

All preterm neonates born <34 weeks’ gestation were eligible for RPS. Those with major congenital malformations, chromosomal aberrations, and contraindications for enteral feeding, and those where no informed consent was available were excluded.

### Primary outcome

Incidence of NEC ≥ Stage II [36].

### Secondary outcomes

All-cause mortality, ‘NEC ≥ Stage II or all-cause mortality’, blood culture positive late onset sepsis (LOS) after 72 hours of life, and postnatal age at full feeds (150 ml/kg/day).

All outcomes were monitored till discharge or death during initial hospitalisation.

The diagnosis of pneumatosis intestinalis by the attending neonatologist was verified independently by the radiologist on call. In case of disagreement, consensus was reached by group discussion between the neonatal and radiology team during the weekly grand rounds and subsequently the final diagnosis was used for coding in the database.

### Probiotic protocol

When ready for enteral feeds, neonates were supplemented with the freshly reconstituted contents of the probiotic sachets every day, and continued until the corrected age 37 weeks [35]. Breast milk (first choice) or sterile water for injection was used for reconstitution of the dry powder in the 1gram sachets. The dose was 3×10^9^ (3 billion) cfu/day (1.5 ml of the reconstituted solution), given as a single dose via the orogastric feeding tube. For neonates <28 weeks the daily dose was 1.5×10^9^ cfu/day until reaching feeds of 50 ml/kg/day. It was then increased to 3×10^9^ cfu/day. The probiotic supplementation was stopped when feeds were stopped by the attending neonatologist for indications such as sepsis and NEC. Safety was assessed by monitoring for blood culture positive sepsis by *B*. *breve* M-16V. The automated blood culture system used by our laboratory detects *B*. *breve* M-16 V within the routine 5 day incubation period. We used the BACTEC^™^ PEDS PLUS^™^/F Medium blood culture vials with incubation monitored in the Bactec 9120 system [38]. Adherence to probiotic protocol was ensured by checking the medication charts of all eligible neonates.

### Sample size estimation

Since our baseline incidence of NEC ≥ stage II was 3–4%, a total sample of 1800, or ~2 years of data from before and after introducing RPS, based on annual admission rates of ~500, was considered to be adequate to achieve 80% power to observe an effect size of 60% with an alpha error of 0.05. The desired effect size was based on the previous systematic reviews [16–21].

Study infants were identified by interrogating our Neonatal Database. Clinical details of all admissions to our unit are entered into this database by trained, dedicated staff. The database is used by the Australia and New Zealand Neonatal Network (ANZNN), for publishing annual reports [39]. The ANZNN conducts regular audits to ensure accuracy of the recorded data.

### Statistical considerations

Descriptive data were summarised using medians, interquartile ranges (IQR) and ranges (R) for continuous outcomes, and frequency distributions for categorical outcomes. Univariate comparisons for continuous data were made using Mann Whitney tests and for categorical data using Chi-square or by using exact inference. The duration of respiratory support measures such as ventilation, continuous positive airway pressure (CPAP) and oxygen was summarised using Kaplan-Meier survival estimates and compared between epochs using the log rank test. Neonatal outcomes of NEC, mortality, LOS and age at full feeds were analysed using multiple logistic regression with adjustment for gestational age <28 weeks and intrauterine growth restriction (IUGR: Birth weight <10^th^ centile for gestation). Characteristics that differed between epochs and other parameters considered to influence neonatal outcomes (e.g. maternal antenatal antibiotics) were also assessed during modelling. The effects of epochs were summarised as unadjusted (OR) and adjusted odds ratios (aOR) with 95% confidence intervals (CI). The analysis was conducted on all neonates <34 weeks’ gestation, and in a subset of neonates <28 weeks who are at a higher risk for NEC. Adjustment for multiple testing was not utilized for the subgroup analysis as insufficient statistical power was considered likely. All tests were two-sided, and a p-value <0.05 was considered statistically significant. The analysis was performed using IBM SPSS 20.0 for Windows (IBM, Armonk, NY) and StatXact 8.0 (Cytel Inc, MA).

### Reporting

The STROBE checklist for reporting observational studies was used [40].

## Results

**Fig 1:**
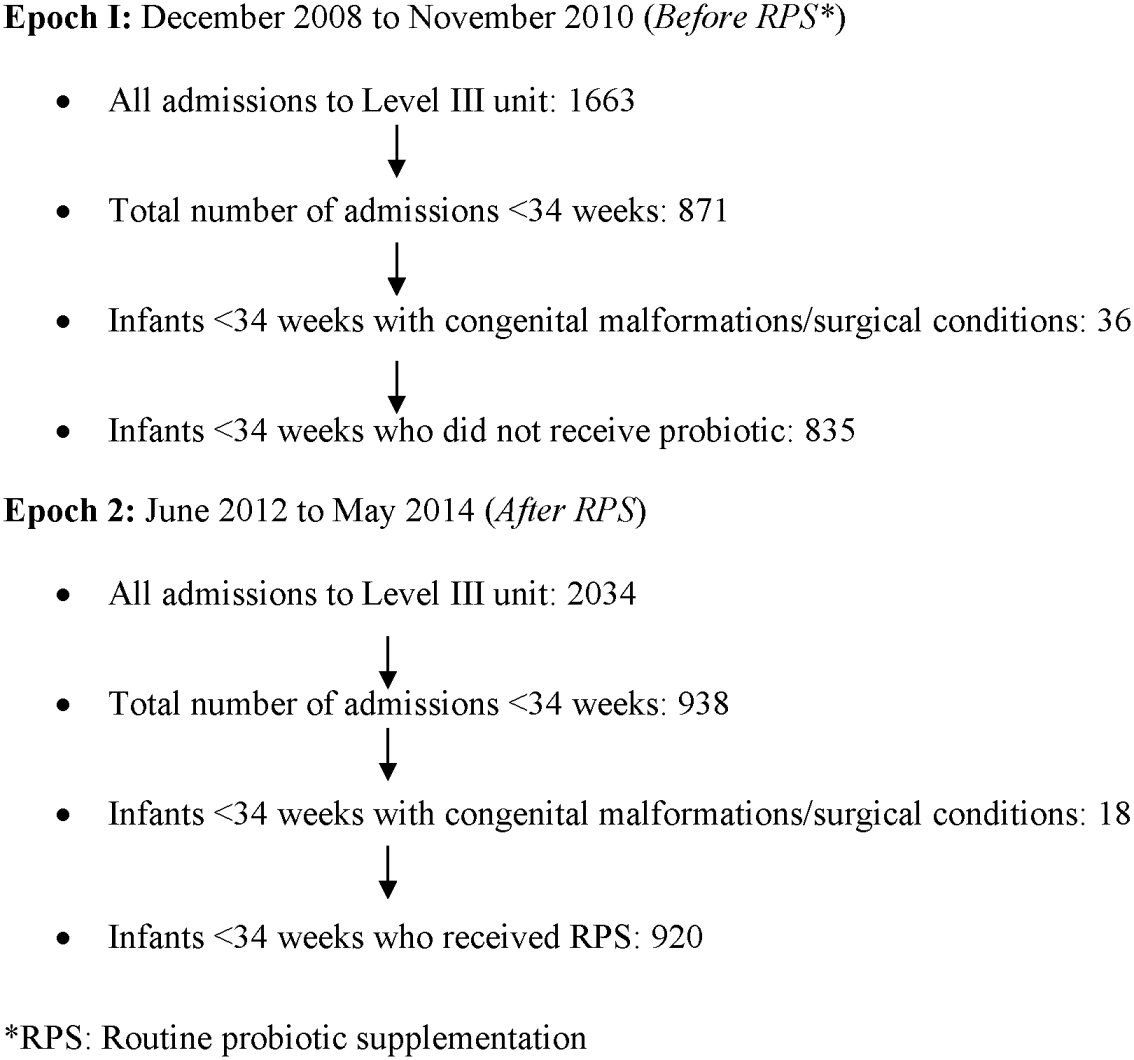
Patient flow diagram.

A total of 1755 preterm neonates born <34 weeks (Epoch I vs. II: 835 vs. 920) were admitted to the nursery over the two epochs (Fig 1). A total of 57/835 (6.8%) and 42/920 (4.6%) infants from Epoch 1 and 2 respectively, were transferred to another hospital for ongoing care. Complete information from all infants (discharged home or transferred to another hospital) was available with no loss to follow up. Their median gestation and birth weight were comparable (Table 1). The frequency of maternal antenatal antibiotic (Erythromycin or Benzyl penicillin) use and gestation at birth <28 weeks was lower in Epoch II (Table 1). Most mothers received antenatal steroids; Epoch I: 717 (93%) vs. Epoch II: 791 (91%): 197 (26%) vs. 193 (22%) single dose, 313 (41%) vs. 320 (37%) complete course, 207 (27%) vs. 278 (32%) >7 days from last dose to delivery (p = 0.021). In Epoch II, there was an increased use of CPAP, oxygen support and oxygen at 36 weeks, and reduced incidence of intraventricular hemorrhage (IVH) (Table 1) [41]. A statistically non-significant increase in the incidence of retinopathy of prematurity (ROP) was noted in Epoch II (Table 1) [42].

**Table 1:** Pregnancy and neonatal characteristics.

	Epoch I N = 835	Epoch II N = 920	
Characteristics	N (%)	N (%)	p-value
Maternal			
**PIH**	157 (19%)	186 (20%)	0.455
**APH**	233 (28%)	228 (25%)	0.138
**Chorioamnionitis**	80 (10%)	109 (12%)	0.126
Antibiotics	478 (57%)	371 (40%)	**<0.001**
Glucocorticoids	717 (93%)	791 (91%)	0.099
**PPROM**	278 (33%)	273 (30%)	0.103
**Inborn**	790 (95%)	864 (94%)	0.531
Gestation (w)*	30 (27–32;23–33)	30 (28–32;23–33)	0.101
**Gestation <28 w**	250 (30%)	220 (24%)	**0.004**
Mode of delivery			
** Vaginal**	**349 (42%)**	366 (40%)	0.402
** Caesarean section**	**486 (58%)**	553 (60%)	
Neonatal			
Birth weight (g)*	1340 (925–1670;293–2980)	1340 (1000–1696;330–2560)	0.145
Male gender	458 (55%)	488 (53%)	0.448
**Apgar <7 at 5 minutes**	157 (19%)	150 (16%)	0.169
**IUGR**	83 (10%)	103 (11%)	0.393
**Respiratory support**			
** Ventilation**	517 (62%)	538 (59%)	0.142
** CPAP**	687 (82%)	800 (87%)	**0.006**
Oxygen	551 (94%)	705 (98%)	**<0.001**
Duration (h)^#^			
Ventilation	27 (11–229)	19 (10–122)	**0.001**
CPAP	128 (28–815)	168 (36–861)	0.444
Oxygen	56 (5–929)	58 (5–633)	0.349
**Oxygen 36 weeks**	**85 (10%)**	148 (16%)	**<0.001**
**PDA**	**264 (32%)**	272 (30%)	0.351
Treated	**157 (60%)**	180 (66%)	0.108
IVH Grade III-IV	**43 (5%)**	25 (3%)	**0.009**
ROP Stage III-IV	**14 (2%)**	25 (3%)	0.095
Early onset sepsis	**15 (2%)**	13 (1.5%)	0.522
Received formula	**34 (4%)**	36 (4%)	0.865
Length of nursery stay (d) ^#^	**36 (21–64)**	37 (19–63)	0.785
Discharge weight* (g)	**2093 (1831–2439;545–4465)**	2280 (1905–2784;606–5580)	**<0.001**

*Median (IQR, range)

^#^Median, IQR, Kaplan-Meier survival estimatesPIH: Pregnancy induced hypertension, APH: Antepartum hemorrhage, PPROM: Preterm pre-labour rupture of membranes, IUGR: Intrauterine growth restriction, CPAP: Continuous positive airway pressure, PDA: Patent ductus arteriosus, IVH: Intraventricular hemorrhage, ROP: Retinopathy of prematurity

### Outcomes for neonates <34 weeks

NEC ≥ Stage II was significantly reduced in Epoch II after adjustment for gestation <28 weeks, IUGR, maternal antenatal antibiotics, CPAP and oxygen support (Table 2). In Epochs I and II respectively, there were 25 (3%) NEC ≥ Stage II (10 Stage III including 3 surgical cases) and 12 (1%) NEC ≥ Stage II (5 Stage III with no surgical cases) (p<0.001), of which 17 (68%) and 11 (92%) survived to discharge (p = 0.220).

**Table 2:** Outcomes for neonates <34 weeks.

<34 weeks	Epoch I N = 835	Epoch II N = 920	Unadjusted OR (CI)	Adjusted aOR (CI)	p-value
NEC^1^	25 (3%)	12 (1%)	0.43 (0.21–0.86)	0.43 (0.21–0.87)	0.019
Mortality^2^	56 (7%)	37 (4%)	0.58 (0.38–0.89)	0.58 (0.31–1.06)	0.078
NEC/Mortality^2^	73 (9%)	48 (5%)	0.57 (0.39–0.84)	0.53 (0.32–0.88)	0.014
Late onset sepsis^3^	**120 (14%)**	82 (9%)	0.58 (0.43–0.79)	0.57 (0.42–0.78)	0.001
Age at full feeds^4^ (d)	**10 (7–17)**	7 (5–12)	HR: 1.61 (1.46–1.78)	HR: 1.79 (1.62–1.98)	<0.001

^1^Adjusted for gestation<28w, IUGR, CPAP, oxygen support

^2^Adjusted for gestation<28w, IUGR, CPAP, oxygen support, maternal antenatal antibiotics, early onset sepsis, IVH

^3^Adjusted for gestation<28w, CPAP, oxygen support, PDA

^4^Data represents median (IQR) Kaplan-Meier estimates, hazard ratios (HR) and 95% confidence intervals (CI) from Cox Hazard regression modelling, adjusted for gestation<28w, IUGR, oxygen support, IVH, PDA

The composite outcome of ‘NEC≥ Stage II or all-cause mortality’ was significantly reduced in Epoch II, but not all-cause mortality as an individual outcome (Table 2). There were 56 and 37 deaths in Epochs I and II, of which 25 (45%) and 15 (38%) were within 72 hours of birth respectively.

Postnatal age at full feeds and incidence of LOS were reduced in Epoch II (Table 2).

The incidence of patent ductus arteriosus (PDA: Left atrium to aortic root ratio >1.4 or ductal diameter >1.5 mm with a left to right shunt) requiring treatment was not significantly different between epochs (Table 1).

### Outcomes for neonates <28 weeks

There were 21% and 14% neonates <28 weeks gestation with ‘NEC≥ Stage II or all-cause mortality’ in Epochs I and II respectively (Table 3). On Univariate analysis, there was a significant reduction in ‘NEC ≥ Stage II or all—cause mortality’ in Epoch II (OR 0.60, CI 0.37–0.98, p = 0.042), but the reduction was no longer significant after adjustment (Table 3). The individual outcomes of NEC ≥ Stage II and all-cause mortality did not significantly differ between epochs. Postnatal age at full feeds and LOS were reduced in Epoch II (Table 3).

**Table 3:** Outcomes for neonates <28 weeks.

<28 weeks	Epoch I N = 250	Epoch II N = 220	Unadjusted OR (CI)	Adjusted aOR (CI)	p-value
NEC^1^	16 (6%)	7 (3%)	0.48 (0.19–1.19)	0.51 (0.20–1.27)	0.148
Mortality^2^	42 (17%)	24 (11%)	0.61 (0.35–1.04)	0.63 (0.28–1.41)	0.258
NEC/Mortality^2^	52 (21%)	30 (14%)	0.60 (0.37–0.98)	0.59 (0.29–1.18)	0.135
Late onset sepsis^3^	**79 (32%)**	44 (20%)	0.54 (0.35–0.83)	0.53 (0.35–0.82)	0.004
Age at full feeds^4^ (d)	**20 (15–27)**	13 (10–17)	HR 2.23(1.81–2.73)	HR 2.44 (1.97–3.01)	<0.001

^1^Adjusted for CPAP

^2^Adjusted for EOS, CPAP, IVH

^3^ Adjusted for GA, CPAP

^4^ Data represents median (IQR) Kaplan-Meier estimates, hazard ratios (HR) and 95% confidence intervals (CI) from Cox Hazard regression modelling, adjusted for GA, IUGR, CPAP, oxygen support

### Safety

There were no adverse effects including probiotic sepsis and abdominal distension, vomiting, and diarrhea needing cessation of the supplementation.

## Discussion

Our results indicate that RPS with *B*. *breve* M-16V was associated with lower incidence of NEC ≥ Stage II in preterm VLBW neonates born <34 weeks. The incidence of NEC ≥ Stage II was lower but not statistically significant in those born <28 weeks, probably because of the small numbers. Using the baseline rate of NEC (6%) in our study, a total sample of 1000, or the equivalent of about 5 years data before and after introducing RPS would be needed (100–125 admissions/year), to detect the desired effect size in neonates <28 weeks.

The benefit of RPS occurred in presence of high rates of breastmilk feeding in our unit that is supported by a human milk bank since 2006, and the low baseline incidence of ≥ Stage II NEC that has remained stable over years. Since 2004 we have adopted a standardised feeding protocol for preterm neonates. To our knowledge no significant changes in clinical practices have taken place over the pre and post RPS study period. The risk of bias in our results is minimised by multiple logistic regression controlling for confounders such as gestation and antenatal maternal antibiotics. Our results are supported by **Satoh et al** who also used *B*. *breve* M-16V for RPS in preterm neonates [43]. Results of RPS are important for assessing benefits of probiotics in real life situation as RCTs may underestimate the effects of probiotics due to cross colonisation of the control group neonates by as much as 44% [44–46].

The results of the multicentre RCT from UK (**PiPS**) are in contrast with benefits of RPS with *B*. *breve* M-16V in preterm infants [47]. This adequately powered (n = 1310) showed no improvement in any of the primary outcomes (NEC or LOS or mortality) in preterm infants <31 weeks gestation supplemented with *B*. *breve* BBG-001 or placebo [47]. There was no probiotic sepsis, further supporting the safety of probiotics in preterm infants. The reasons for the negative results include the 49% cross-colonisation of the placebo arm infants and an inadequate dose towards the end of the shelf life of the product due to loss of viable bacteria. We have discussed these issues in detail elsewhere.

Comparing our results with those from comparable units is important. **Janvier et al** have reported their cohort study in very preterm neonates [48]. All neonates <32 weeks' gestation received RPS with 0.5 g of a mixture of four bifidobacteria (*B*. *breve*, *bifidum*, *infantis*, *and longum*) and *Lactobacillus rhamnosus* HA-111 (2x10^9^ cfu/day), starting with the first feed, and continued until reaching 34 weeks. Data from the first 17 months of RPS (n = 294) were compared with those from previous 17 months without RPS (n = 317). RPS was associated with a reduction in NEC ≥ Stage II (from 9.8% to 5.4%, p < .02), a non-significant decrease in death (9.8% to 6.8%), and a significant reduction in the combined outcome of ‘death or NEC’ (from 17% to 10.5%, p < .05). The improvements [OR (95% CI)] remained significant after adjustment for gestation, IUGR, and sex [NEC: 0.51 (0.26–0.98); Death or NEC: 0.56 (0.33–0.93)]. RPS had no effect on LOS. Neonates with birth weight <1001 grams showed similar percentage reductions in NEC [Pre-RPS: 18 (17%) vs. RPS: 10 (10%)] and the combined outcome of death and NEC [Pre-RPS: 38 (35%) vs. RPS: 22 (22%)] but the numbers (Pre-RPS:109; RPS: 96) were small to reach statistical significance [48]. **Repa et al** have reported that probiotics may not overcome the adverse effects of formula feeding and that their benefits occur in breast milk fed preterm neonates at high risk of NEC [49]. VLBW neonates receiving RPS with a mixture of lactobacilli and bifidobacteria (2010–2012) were prospectively followed. Neonates from 2008 to 2009 without RPS served as controls. RPS had no significant impact on NEC [Controls: 24/233 (10.3%); RPS: 16/230 (7%); p = 0.2]. However, NEC was significantly reduced in RPS group neonates fed any breast milk [20/179 (11.2%) vs. 10/183 (5.5%); p = 0.027]. RPS was ineffective in those on exclusive formula feeding [4/54 (7.4%) vs. 6/44 (13.6%); p = 0.345]. Occurrence of severe NEC (Stage IIIb), time to full feeds, and gastric residuals were similar [49]. **Hartel et al** have reported a cohort of VLBW neonates stratified to prophylactic use of *Lactobacillus acidophilus/B*. *infantis* [50]. Within the observational period (1/9/2010-31/12/2012, n = 5351) participating centers were categorized into 3 groups based on their choice of probiotic use: (1) no prophylactic use; (2 a/b) changing from being nonuser to user during observational period; and (3) use before start of observation. In a multivariable logistic regression analysis, probiotics were protective for NEC surgery (OR: 0.58, 95% CI: 0.37–0.91; p = .017), any abdominal surgery (OR: 0.7, 95% CI: 0.51–0.95; p = .02), and the combined outcome ‘abdominal surgery or death’ (OR: 0.43; 95% CI: 0.33–0.56; p < .001). These findings are important considering the health burden of stage II NEC is primarily related to its progression to stage III [50]. Selection of ‘any abdominal surgery’ as the outcome minimises the risk of bias due to misclassification of spontaneous intestinal perforation as NEC. **Olsen et al** have recently reported a systematic review of observational studies reporting on RPS in preterm neonates [34]. Meta-analysis of data from 12 studies (Prophylactic probiotics: 5,144 vs. Controls: 5,656) showed a significantly decreased incidence of NEC (RR: 0.55, 95% CI: 0.39–0.78; p = 0.0006) and mortality (RR: 0.72, 95% CI: 0.61–0.85; p<0.0001). Late onset sepsis did not differ significantly between the two groups (RR: 0.86, 95% CI: 0.74–1.00; p = 0.05). The effect sizes were similar to findings in meta-analyses of RCTs. There were no adverse events including probiotic sepsis [34].

The **strengths** of our study include its large sample size, use of multivariate regression analysis, benefits of RPS in a setting with low baseline incidence of NEC and donor milk bank, and use of STROBE guidelines for reporting. The **limitation** is its retrospective design which makes it difficult to control for all confounders.

In summary our results indicate that RPS with *B*. *breve* M-16V was associated with significant reduction in ≥ Stage II NEC in preterm VLBW neonates. Caution is warranted in generalising these results considering the variations in patient demographics and clinical practices across units. However, the report by Olsen et al is reassuring in this context [34]. The importance of probiotic quality control cannot be overemphasised considering the report of fatal Mucormycosis in a preterm neonate following the use of a contaminated probiotic product [51].
